# Supplementary material for: A positive feedback loop between mTORC1 and cathelicidin promotes skin inflammation in rosacea
Source: EMBO Mol Med. 2021 Mar 18;13(5):e13560. doi: 10.15252/emmm.202013560 (PMC8103105; doi:10.15252/emmm.202013560)
Supplement: Supplementary file 1 — Appendix [file EMMM-13-e13560-s002.pdf]

Appendix

Table of contents:

Appendix Figure S1. mTORC1 signaling is hyperactivated in the lesional skin of rosacea.

Appendix Figure S2. Deficiency of mTORC1 suppresses rosacea development.

Appendix Figure S3. Hyperactivation of mTORC1 aggravates rosacea development.

Appendix Figure S4. mTORC1 regulates cathelicidin LL37 through a positive feedback circuit.

Appendix Figure S5. Cathelicidin LL37 stimulates mTORC1 via binding to TLR2.

Appendix Figure S6. Cathelicidin LL37 induces NF-κB signaling via mTORC1 in keratinocytes.

Appendix Figure S7. Cathelicidin LL37 induces chemokines and cytokines via mTORC1 in keratinocytes.

Appendix Table S1. Information of patients and healthy individuals skin samples.

Appendix Table S2. List of qPCR primers in this study.

Appendix Table S3. CEA and IGA score and score change for rosacea patients after topical rapamycin treatment.

Appendix Table S4. Summary of significant P values for Main and appendix figures.

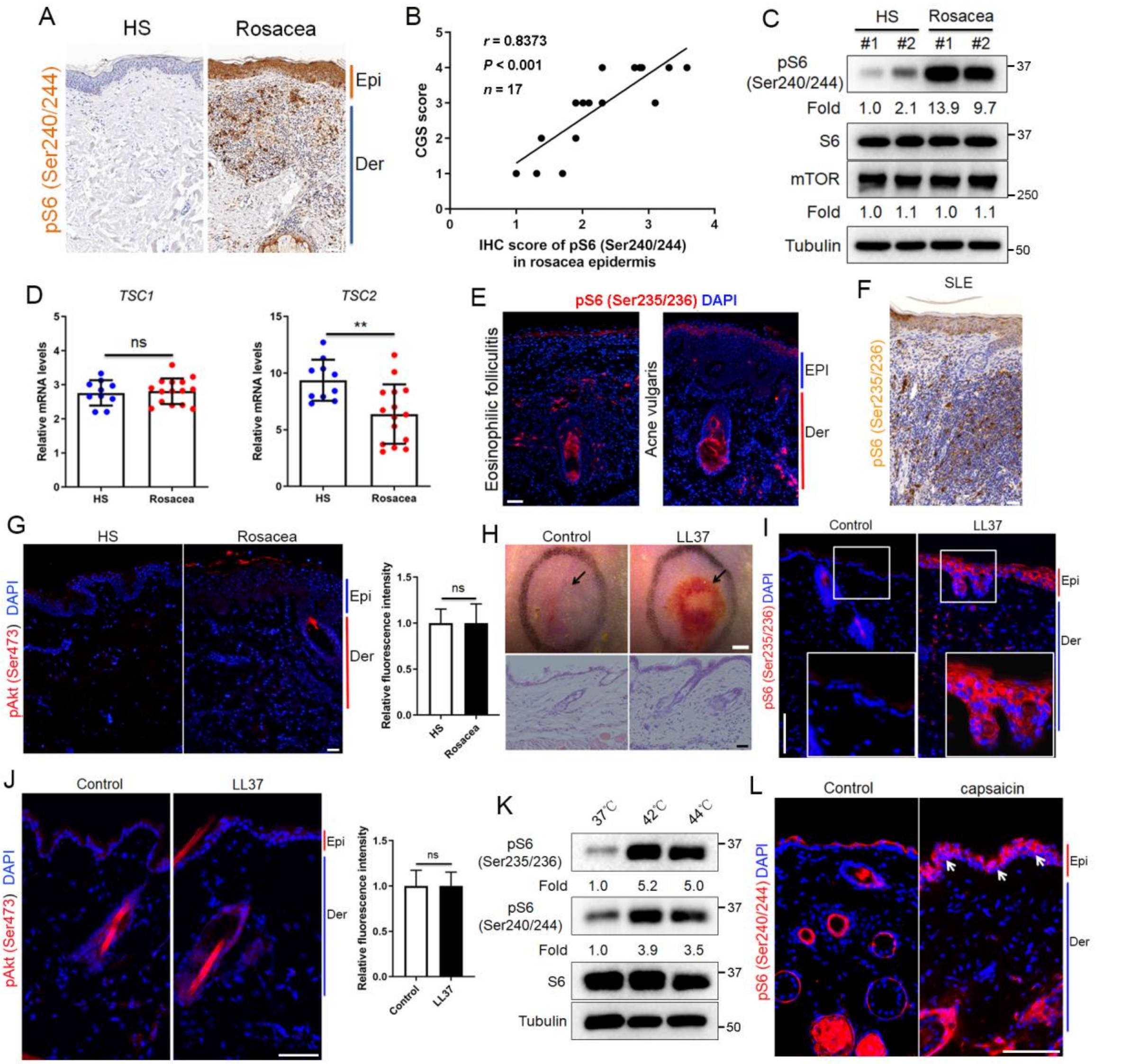

Appendix Figure S1. mTORC1 signaling is hyperactivated in the lesional skin of rosacea.

(**A**) Immunohistochemistry of pS6 (Ser240/244) on skin sections from healthy individuals (HS) and patients with rosacea. Scale bar: 50 μm. (**B**) Correlation of pS6 (Ser240/244) expression (from IHC) in the epidermis of human rosacea lesions (n = 17) with the Clinician's Global Severity (CGS) scores. (**C**) Immunoblotting analysis of pS6 (Ser240/244), S6 and mTOR in skin samples from HS and rosacea patients. pS6 and mTOR protein levels were analyzed relative to total S6 and Tubulin respectively. Tubulin is the loading control. (**D**) The mRNA expression levels of *TSC1* and *TSC2* in skin lesions from healthy individuals (n = 10) and patients with rosacea (n = 15). (**E**) Immunostaining of pS6 on skin sections from patients with acne vulgaris or eosinophilic folliculitis. Scale bar: 50 μm. (**F**) IHC of pS6 on skin sections from patients with lupus erythematosus. Scale bar: 50 μm. (**G**) Immunostaining of p-Akt (Ser473) on skin sections from healthy

individuals and patients with rosacea. Right panel, the quantification of relative fluorescence intensity for pAkt (Ser473) in HS and rosacea patients skin sections (n=5 for each group). Scale bar: 50 µm. (**H**) Top: The back skins of BALB/c WT mice were intradermally injected with LL37 or control vehicle. Scale bar: 2 mm. Bottom: HE staining of lesional skin sections. Scale bar: 50 µm. (**I**) Immunostaining of pS6 (Ser235/236) in skin lesions from control and LL37-induced mice. Bottom right panels, magnified images of boxed areas. Scale bar: 50 µm. (**J**) Immunostaining of p-Akt (Ser473) on skin sections from control and LL37-induced mice. Right panel, the quantification of relative fluorescence intensity for pAkt (Ser473) (n=5 for each group). Scale bar: 50 µm. (**K**) Immunoblotting analysis of pS6 and total S6 in cell lysates from primary human keratinocytes stimulated with heat shock (37 – 44 ℃) for 2 h. pS6 protein levels were analyzed relative to total S6. (**L**) Immunostaining of pS6 (Ser240/244) in skin lesions from control and LL37-induced mice topically applied with 0.1% capsaicin or placebo cream (n=6 for each group). DAPI staining (blue) indicates nuclear localization. Scale bar: 50 µm.

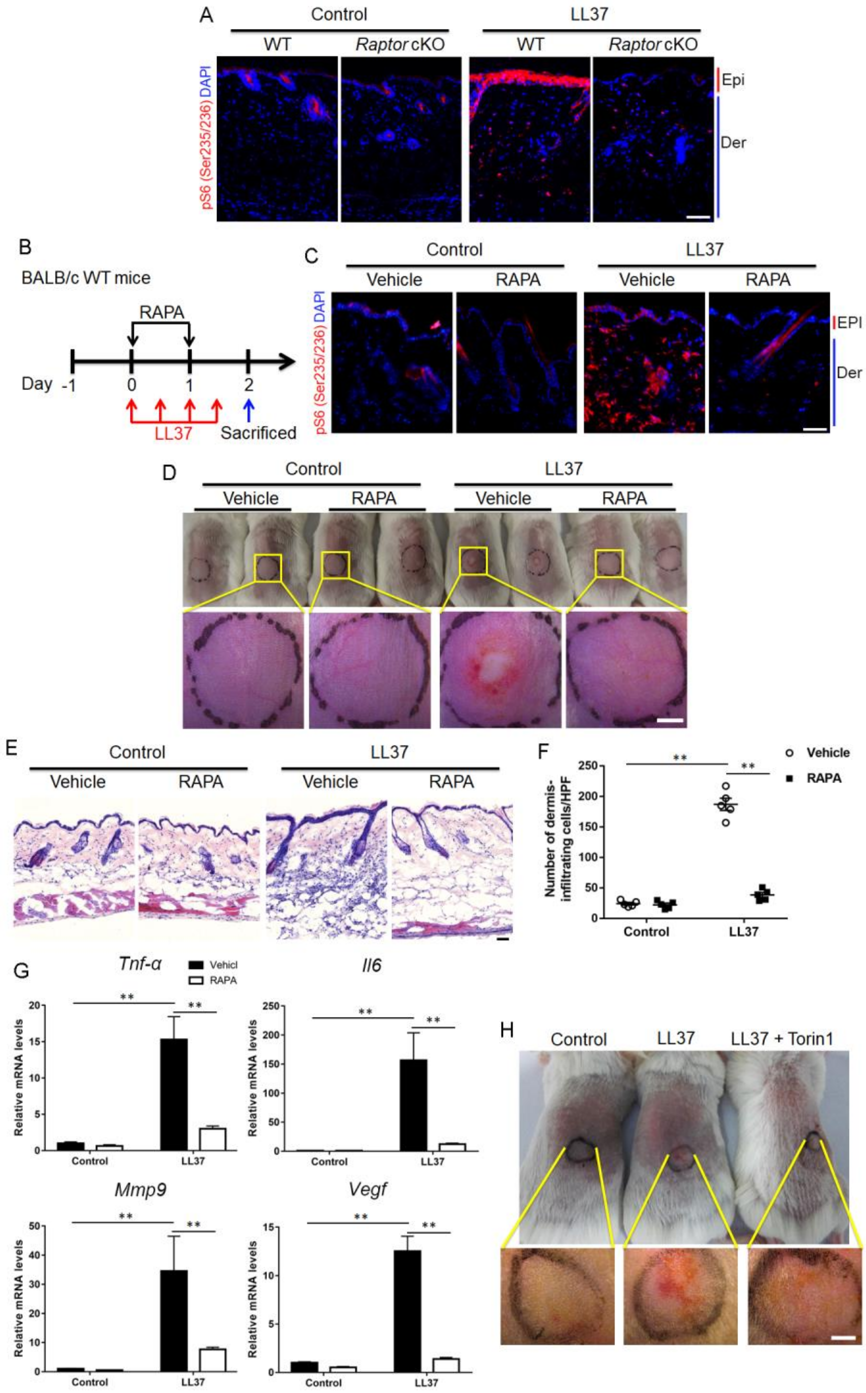

Appendix Figure S2. Deficiency of mTORC1 suppresses rosacea development.

(**A**) Immunostaining of pS6 in skin sections from WT and *Raptor* cKO mice injected with LL37 or control vehicle (n=6). Scale bar: 50 μm. (**B**) Schematic diagram of intraperitoneal administration of rapamycin (RAPA) on day 0 and 1 during the intradermal injection of LL37 in mice (BALB/c). 6-8 mice in each group were sacrificed on day 2 to conduct subsequent experiments. (**C**) Immunostaining of pS6 in skin sections from LL37-injected mice administrated with RAPA or vehicle. Scale bar: 50 μm. (**D**) The back skins of

mice treated with RAPA or vehicle, were intradermally injected with LL37. Images were taken 48 h after the first LL37 injection. Below panels, magnified images of yellow boxed areas. Scale bar: 2 mm. (**E**) HE staining of lesional skin sections from LL37-treated mice injected with RAPA or vehicle. Scale bar: 50 µm. (**F**) Dermal infiltrating cells were quantified (n = 5). (**G**) The mRNA expression levels of *Tnf-α, Il6, Mmp9* and *Vegf* in skin lesions (n = 6-8). (**H**) The back skins of mice treated with torin1 or vehicle, were intradermally injected with LL37 (n=5 for each group). Images were taken 48 h after the first LL37 injection. Below panels, magnified images of black circled areas. Scale bar: 2 mm. All results are representative of at least 3 independent experiments. Data represent the mean ± SEM. **P < 0.01. 1-way ANOVA with Bonferroni's post hoc test was used.

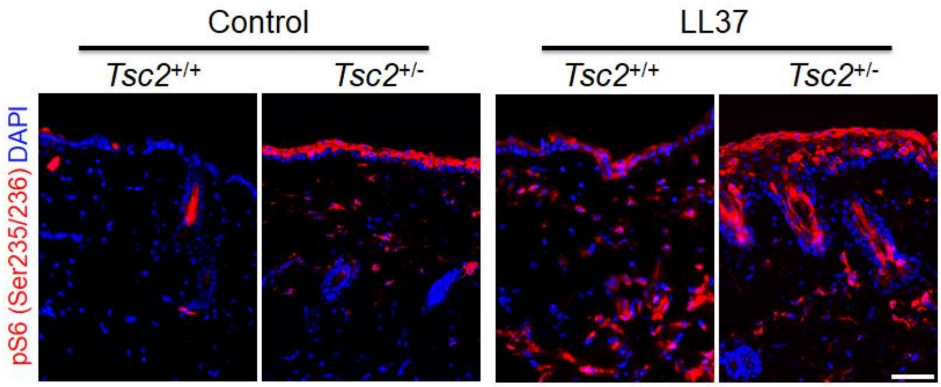

Appendix Figure S3. Hyperactivation of mTORC1 aggravates rosacea development.

Immunostaining of pS6 in lesional skin sections from *Tsc2*$^{+/+}$ and *Tsc2*$^{+/-}$ mice injected with LL37 or control vehicle. DAPI staining (blue) indicates nuclear localization. Scale bar: 50 µm.

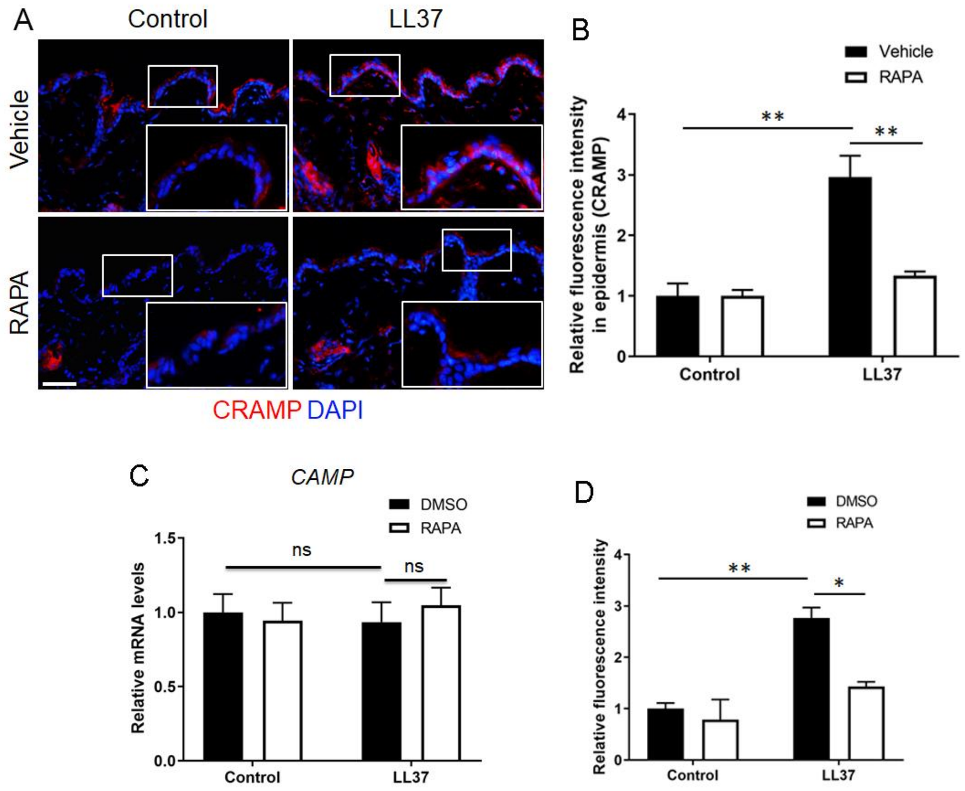

Appendix Figure S4. mTORC1 regulates cathelicidin LL37 through a positive feedback circuit.

(**A**) Immunostaining of mouse CRAMP in skin sections from LL37-injected mice administrated with RAPA or vehicle. DAPI staining (blue) indicates nuclear localization. Scale bar: 50 µm. (**B**) Quantification of relative fluorescence intensity for CRAMP in epidermis (n = 6). (**C**) mRNA expression levels of *CAMP* in primary human keratinocytes treated with LL37 ± RAPA. (**D**) Quantification of relative fluorescence intensity of cathelicidin (related to Fig 4G). All results are representative of at least 3 independent experiments. Data represent the mean ± SEM. *P < 0.05, **P < 0.01. “ns” indicates no statistical significance. 1-way ANOVA with Bonferroni’s post hoc test was used.

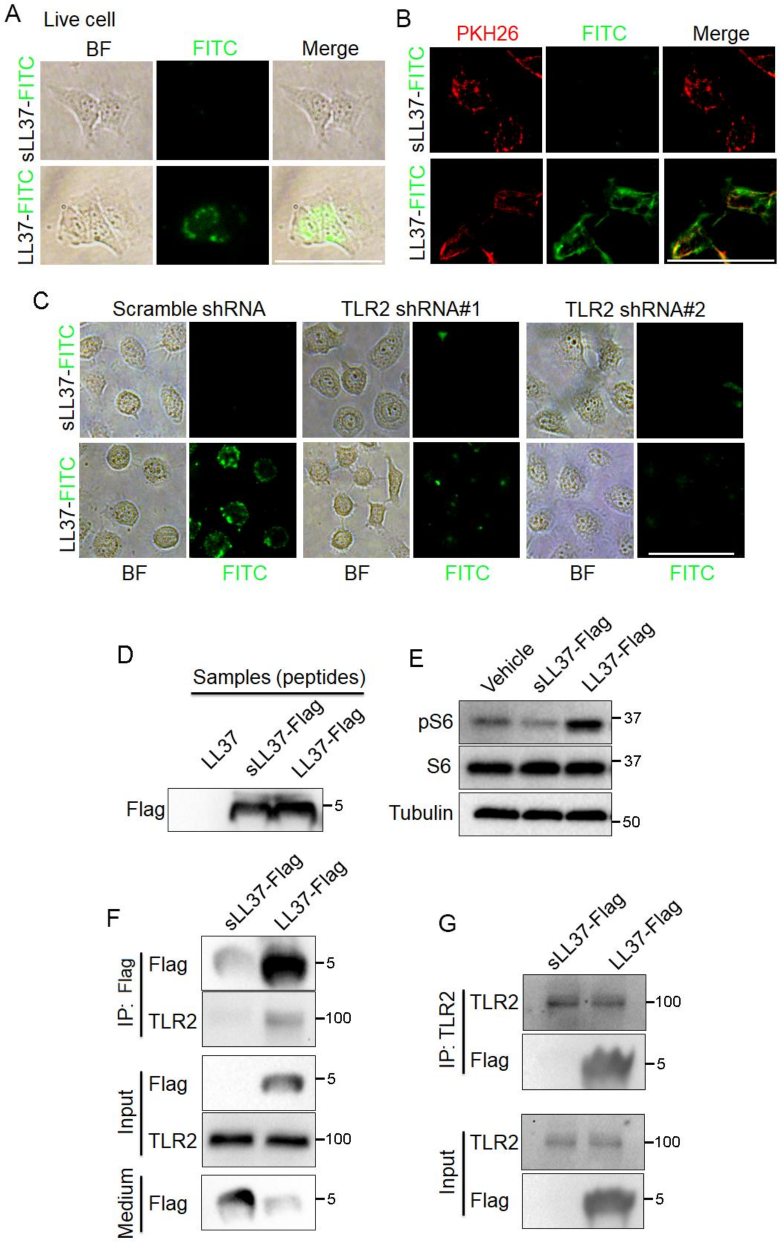

Appendix Figure S5. Cathelicidin LL37 stimulates mTORC1 via binding to TLR2.

(**A**) Representative images showing cellular localization of LL37 or sLL37 analyzed by fluorescent microscope in live HaCaT keratinocytes treated with FITC-labeled LL37 or sLL37 (4 μM) for 30 min followed by PBS washed. BF, bright field. Scale bar: 50 μm. (**B**) Representative images showing membrane localization of LL37 or sLL37 in live HaCaT keratinocytes treated with FITC-labeled LL37 / sLL37 and PKH26 for 30 min. Scale bar: 50 μm. (**C**) HaCaT keratinocytes expressing TLR2 shRNAs or scramble shRNA were treated with FITC-labeled LL37 or sLL37 for 30 min followed by PBS washed, then live cells were analyzed by fluorescent microscope. Scale bar: 50 μm. (**D**) Immunoblotting analysis of flag in peptide

samples (LL37, sLL37-flag and LL37-flag). (**E**) Immunoblotting of pS6 (Ser235/236) and total S6 in cell lysates from HaCaT keratinocytes treated with sLL37-flag and LL37-flag for 2 h. (**F** and **G**) HaCaT keratinocyte cells were incubated with sLL37-flag or LL37-flag for 1 h. Cell lysates were immunoprecipitated with anti-flag or anti-TLR2 antibodies, showing an interaction between LL37 and TLR2. Medium, the medium of HaCaT keratinocytes treated with sLL37-flag or LL37-flag. All results are representative of at least 3 independent experiments.

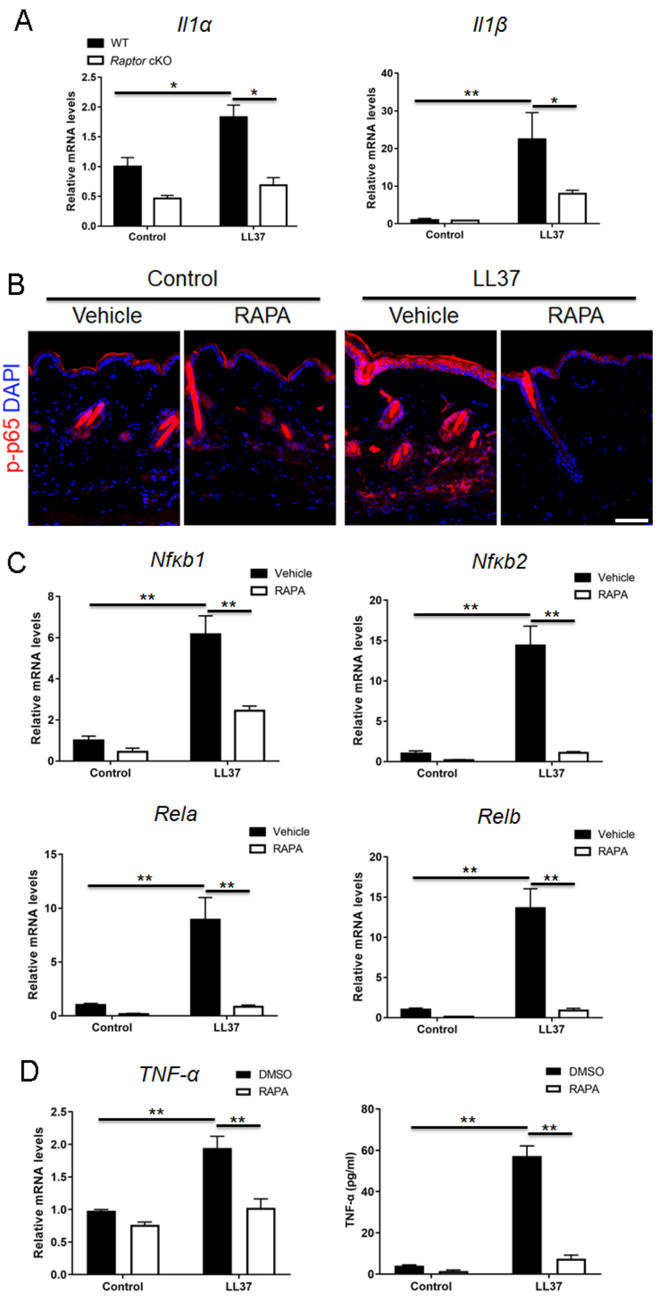

Appendix Figure S6. Cathelicidin LL37 induces NF-κB signaling via mTORC1 in keratinocytes.

(**A**) The mRNA expression levels of NF-κB target genes (*Il1α* and *Il1β*) in WT or *Raptor* cKO mice skin injected with LL37 or control vehicle (n=6). (**B**) Immunostaining of p-p65 in skin sections from LL37-injected mice administrated with RAPA or vehicle. Scale bar: 50 μm. (**C**) The mRNA expression levels of NF-κB family of transcription factors (Nfκb1, Nfκb1, Rela and Relb) in skin lesions (n=6). (**D**) The mRNA levels (left) and medium protein levels (right) of TNF-α in primary human keratinocytes treated with LL37 ± RAPA. All results are representative of at least 3 independent experiments. Data represent the mean ± SEM. **P < 0.01. 1-way ANOVA with Bonferroni's post hoc test was used.

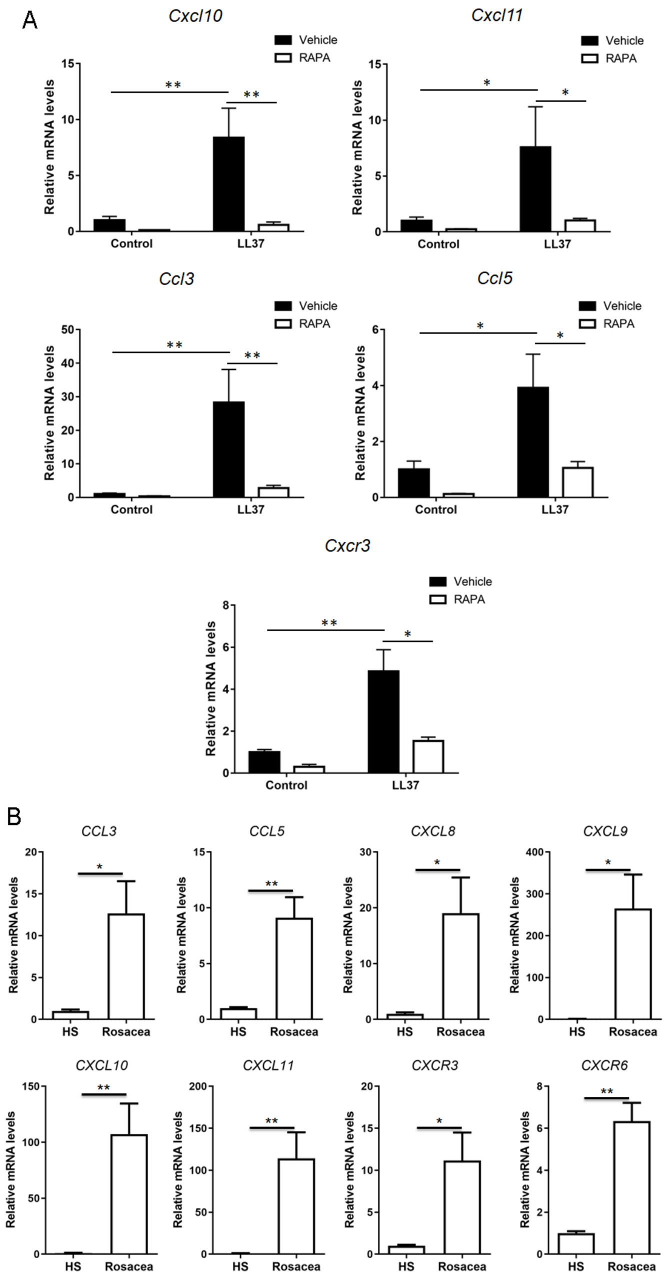

Appendix Figure S7. Cathelicidin LL37 induces chemokines and cytokines via mTORC1 in keratinocytes.

(**A**) The mRNA expression levels of mouse chemokines (Cxcl10, Cxcl11, Ccl3, Ccl5 and Cxcr3) in skin lesions (n=6). (**B**) The mRNA expression levels of rosacea-associated chemokines and cytokines in HS (n=10) and rosacea patient (n=15) skins detected by qPCR. All results are representative of at least 3 independent experiments. Data represent the mean ± SEM. *P < 0.05, **P < 0.01. two-tailed unpaired Student's t-test (B) or 1-way ANOVA with Bonferroni's post hoc test (A) was used.

Appendix Table S1. Information of patients and healthy individuals skin samples.

Sample ID	Sample classification	Age/Gender	CGS score	Application
1	HS	27/Female	Not applicable	RNA-sequencing
2	HS	40/Female	Not applicable	RNA-sequencing
3	HS	23/Female	Not applicable	RNA-sequencing
4	HS	27/Female	Not applicable	RNA-sequencing
5	HS	30/Female	Not applicable	RNA-sequencing
6	HS	21/Female	Not applicable	RNA-sequencing
7	HS	31/Female	Not applicable	RNA-sequencing
8	HS	49/Female	Not applicable	RNA-sequencing
9	HS	27/Female	Not applicable	RNA-sequencing
10	HS	46/Female	Not applicable	RNA-sequencing
11	HS	28/Female	Not applicable	RT-qPCR
12	HS	42/Female	Not applicable	RT-qPCR
13	HS	31/Female	Not applicable	RT-qPCR
14	HS	27/Female	Not applicable	RT-qPCR
15	HS	23/Female	Not applicable	RT-qPCR
16	HS	32/Female	Not applicable	RT-qPCR
17	HS	29/Female	Not applicable	RT-qPCR
18	HS	33/Female	Not applicable	RT-qPCR
19	HS	21/Female	Not applicable	RT-qPCR
20	HS	40/Female	Not applicable	RT-qPCR
21	HS	22/Female	Not applicable	IHC/IF
22	HS	47/Female	Not applicable	IHC/IF
23	HS	30/Female	Not applicable	IHC/IF
24	HS	32/Female	Not applicable	IHC/IF
25	HS	27/Female	Not applicable	IHC/IF
26	HS	41/Female	Not applicable	IHC/IF
27	HS	40/Female	Not applicable	IHC/IF
28	HS	21/Female	Not applicable	IHC/IF
29	Rosacea	45/Female	3	RNA-sequencing
30	Rosacea	45/Female	2	RNA-sequencing
31	Rosacea	50/Female	3	RNA-sequencing
32	Rosacea	36/Female	3	RNA-sequencing
33	Rosacea	42/Female	4	RNA-sequencing
34	Rosacea	30/Female	2	RNA-sequencing
35	Rosacea	36/Female	2	RNA-sequencing
36	Rosacea	24/Female	4	RNA-sequencing
37	Rosacea	20/Female	1	RNA-sequencing
38	Rosacea	29/Female	3	RNA-sequencing
39	Rosacea	46/Female	3	RT-qPCR

40	Rosacea	25/Female	2	RT-qPCR
41	Rosacea	38/Female	4	RT-qPCR
42	Rosacea	48/Female	1	RT-qPCR
43	Rosacea	29/Female	3	RT-qPCR
44	Rosacea	26/Female	3	IHC/IF
45	Rosacea	34/Female	2	IHC/IF
46	Rosacea	23/Female	4	IHC/IF
47	Rosacea	26/Female	3	IHC/IF
48	Rosacea	37/Female	2	IHC/IF
49	Rosacea	49/Female	4	IHC/IF
50	Rosacea	42/Female	4	IHC/IF
51	Rosacea	29/Female	3	IHC/IF
52	Rosacea	33/Female	4	IHC/IF
53	Rosacea	36/Female	1	IHC/IF
54	Rosacea	34/Female	3	IHC/IF
55	Rosacea	43/Female	3	IHC/IF
56	Rosacea	35/Female	1	IHC/IF
57	Rosacea	29/Female	4	IHC/IF
58	Rosacea	41/Female	1	IHC/IF
59	Rosacea	24/Female	4	IHC/IF
60	Rosacea	30/Female	3	IHC/IF
61	Acne vulgaris	31/Female	Typical	IHC/IF
62	Acne vulgaris	27/Female	Typical	IHC/IF
63	Acne vulgaris	35/Female	Typical	IHC/IF
64	Eosinophilic folliculitis	32/Female	Typical	IHC/IF
65	Eosinophilic folliculitis	22/Female	Typical	IHC/IF
66	Eosinophilic folliculitis	29/Female	Typical	IHC/IF
67	Lupus erythematosus	40/Female	Typical	IHC/IF
68	Lupus erythematosus	28/Female	Typical	IHC/IF
69	Lupus erythematosus	36/Female	Typical	IHC/IF

Appendix Table S2. List of qPCR primers in this study.

Human-*GAPDH*-F	TGTTGCCATCAATGACCCCTT
Human -*GAPDH*-R	CTCCACGACGTACTCAGCG
Human-*CAMP*-F	GGCTGGTGAAGCGGTGTAT
Human -*CAMP*-R	TGGGTACAAGATTCCGCAAAAA
Human-*CXCL8*-F	TTTTGCCAAGGAGTGCTAAAGA
Human -*CXCL8*-R	AACCCTCTGCACCCAGTTTTC
Human- *CXCL9*-F	CCAGTAGTGAGAAAGGGTCGC
Human -*CXCL9*-R	AGGGCTTGGGGCAAATTGTT
Human- *CXCL10*-F	GTGGCATTCAAGGAGTACCTC

Human -*CXCL10*-R	TGATGGCCTTCGATTCTGGATT
Human- *CXCL11*-F	GACGCTGTCTTTGCATAGGC
Human -*CXCL11*-R	GGATTTAGGCATCGTTGTCCTTT
Human- *CCL2*-F	CAGCCAGATGCAATCAATGCC
Human -*CCL2*-R	TGGAATCCTGAACCCACTTCT
Human- *CCL20*-F	TGCTGTACCAAGAGTTTGCTC
Human -*CCL20*-R	CGCACACAGACAACTTTTTCTTT
Human- *IL6*-F	CCTGAACCTTCCAAAGATGGC
Human -*IL6*-R	TTCACCAGGCAAGTCTCCTCA
Human- *TNFα*-F	CCTCTCTCTAATCAGCCCTCTG
Human -*TNFα*-R	GAGGACCTGGGAGTAGATGAG
Human- *MMP9*-F	AGACCTGGGCAGATTCCAAAC
Human -*MMP9*-R	CGGCAAGTCTTCCGAGTAGT
Human- *VEGFA*-F	AGGGCAGAATCATCACGAAGT
Human -*VEGFA*-R	AGGGTCTCGATTGGATGGCA
Human- TSC1-F	CAACAAGCAAATGTCGGGGAG
Human –TSC1-R	CATAGGGCCACGGTCAGAA
Human- TSC2-F	CCAAACCAACAAGCAAAGATTCA
Human –TSC2-R	CACATTCCATGCTCAGTTCTCT
Mouse-*Gapdh*-F	AGGTCGGTGTGAACGGATTTG
Mouse -*Gapdh*-R	TGTAGACCATGTAGTTGAGGTCA
Mouse -*Tnfα*-F	CTGAACTTCGGGGTGATCGG
Mouse -*Tnfα*-R	GGCTTGTCACTCGAATTTTGAGA
Mouse -*Il6*-F	TAGTCCTTCCTACCCCAATTTCC
Mouse -*Il6*-R	TTGGTCCTTAGCCACTCCTTC
Mouse -*Cramp*-F	GCTGTGGCGGTCACTATCAC
Mouse -*Cramp*-R	TGTCTAGGGACTGCTGGTTGA
Mouse -*Nfkb1*-F	ATGGCAGACGATGATCCCTAC
Mouse - *Nfkb1*-R	TGTTGACAGTGGTATTTCTGGTG
Mouse -*Nfkb2*-F	GGCCGGAAGACCTATCCTACT
Mouse -*Nfkb2*-R	CTACAGACACAGCGCACACT
Mouse -*Rela*-F	AGGCTTCTGGGCCTTATGTG
Mouse -*Rela*-R	TGCTTCTCTCGCCAGGAATAC
Mouse -*Relb*-F	CCGTACCTGGTCATCACAGAG
Mouse -*Relb*-R	CAGTCTCGAAGCTCGATGGC
Mouse -*Cxcl10*-F	CCAAGTGCTGCCGTCATTTTC
Mouse -*Cxcl10*-R	GGCTCGCAGGGATGATTTCAA
Mouse -*Cxcl11*-F	GGCTTCCTTATGTTCAAACAGGG
Mouse -*Cxcl11*-R	GCCGTTACTCGGGTAAATTACA
Mouse -*Cxcl12*-F	TGCATCAGTGACGGTAAACCA
Mouse -*Cxcl12*-R	TTCTTCAGCCGTGCAACAATC

Mouse -*Ccl2*-F	TTAAAAACCTGGATCGGAACCAA
Mouse -*Ccl2*-R	GCATTAGCTTCAGATTTACGGGT
Mouse - *Ccl3*-F	TTCTCTGTACCATGACACTCTGC
Mouse - *Ccl3*-R	CGTGGAATCTTCCGGCTGTAG
Mouse -*Ccl5*-F	GCTGCTTTGCCTACCTCTCC
Mouse -*Ccl5*-R	TCGAGTGACAAACACGACTGC
Mouse -*Il1α*-F	CGAAGACTACAGTTCTGCCATT
Mouse -*Il1α*-R	GACGTTTCAGAGGTTCTCAGAG
Mouse -*Il1β*-F	GCAACTGTTCCTGAACTCAACT
Mouse -*Il1β*-R	ATCTTTTGGGGTCCGTCAACT
Mouse -*Mmp9*-F	CTGGACAGCCAGACACTAAAG
Mouse -*Mmp9*-R	CTCGCGGCAAGTCTTCAGAG
Mouse -*Cxcr3*-F	TACCTTGAGGTTAGTGAACGTCA
Mouse -*Cxcr3*-R	CGCTCTCGTTTTCCCCATAATC
Mouse -*Vegf*-F	TATTCAGCGGACTCACCAGC
Mouse -*Vegf*-R	AACCAACCTCCTCAAACCGT

Appendix Table S3. CEA and IGA score and score change for rosacea patients after topical rapamycin treatment.

	CEA			IGA		
	0 W	4 W	Change	0 W	4 W	Change
**Placebo**						
Patient 1	2	2	0	3	3	0
Patient 2	3	3	0	3	3	0
Patient 3	1	2	1	1	1	0
Patient 4	2	2	0	3	3	0
Patient 5	2	2	0	1	1	0
Patient 6	2	2	0	2	2	0
Patient 7	4	3	-1	3	3	0
Patient 8	4	3	-1	4	3	-1
**Rapamycin**						
Patient 1	3	1	-2	3	1	-2
Patient 2	3	2	-1	3	1	-2
Patient 3	3	2	-1	4	2	-2
Patient 4	2	2	0	3	2	-1
Patient 5	2	2	0	3	2	-1
Patient 6	2	1	-1	2	2	0
Patient 7	3	2	-1	2	2	0
Patient 8	2	2	0	2	1	-1
Patient 9	3	2	-1	3	2	-1
Patient 10	3	1	-2	3	1	-2

Appendix Table S4. Summary of significant P values for Main and appendix figures.

Figure 1D	P = 0.018
Figure 1G	P = 0.0019
Figure 2E	WT: LL37vsControl P<0.0001; LL37: Raptor cKOvsWT P<0.0001;
Figure 2F (Tnfa)	WT: LL37vsControl P=0.0007; LL37: Raptor cKOvsWT P=0.007;
Figure 2F (Il6)	WT: LL37vsControl P<0.0001; LL37: Raptor cKOvsWT P=0.0016;
Figure 2F (Mmp9)	WT: LL37vsControl P=0.0077; LL37: Raptor cKOvsWT P=0.00285;
Figure 2F (Vegf)	WT: LL37vsControl P<0.0001; LL37: Raptor cKOvsWT P<0.0001;
Figure 2J	WT: LL37vsControl P<0.0001; LL37: Raptor cKOvsWT P<0.0001;
Figure 3B	**Day 0.5** **P value**<br>WT-PBS vs. WT-LL37 0.9505<br>WT-PBS vs. TSC2-PBS >0.9999<br>WT-PBS vs. TSC2-LL37 <0.0001<br>WT-LL37 vs. TSC2-PBS 0.9590<br>WT-LL37 vs. TSC2-LL37 <0.0001<br>TSC2-PBS vs. TSC2-LL37 <0.0001<br>**Day 2** **P value**<br>WT-PBS vs. WT-LL37 <0.0001<br>WT-PBS vs. TSC2-PBS >0.9999<br>WT-PBS vs. TSC2-LL37 <0.0001<br>WT-LL37 vs. TSC2-PBS <0.0001<br>WT-LL37 vs. TSC2-LL37 <0.0001<br>TSC2-PBS vs. TSC2-LL37 <0.0001<br>**Day 5** **P value**<br>WT-PBS vs. WT-LL37 <0.0001<br>WT-PBS vs. TSC2-PBS >0.9999<br>WT-PBS vs. TSC2-LL37 <0.0001<br>WT-LL37 vs. TSC2-PBS <0.0001<br>WT-LL37 vs. TSC2-LL37 <0.0001<br>TSC2-PBS vs. TSC2-LL37 <0.0001<br>**Day 8** **P value**<br>WT-PBS vs. WT-LL37 0.5044<br>WT-PBS vs. TSC2-PBS >0.9999<br>WT-PBS vs. TSC2-LL37 <0.0001<br>WT-LL37 vs. TSC2-PBS 0.5044<br>WT-LL37 vs. TSC2-LL37 <0.0001

	TSC2-PBS vs. TSC2-LL37 <0.0001<br>**Day 12 P value**<br>WT-PBS vs. WT-LL37 >0.9999<br>WT-PBS vs. TSC2-PBS >0.9999<br>WT-PBS vs. TSC2-LL37 0.0756<br>WT-LL37 vs. TSC2-PBS >0.9999<br>WT-LL37 vs. TSC2-LL37 0.0756<br>TSC2-PBS vs. TSC2-LL37 0.0756
Figure 3C	**Day 0.5 P value**<br>WT-PBS vs. WT-LL37 0.9375<br>WT-PBS vs. TSC2-PBS >0.9999<br>WT-PBS vs. TSC2-LL37 <0.0001<br>WT-LL37 vs. TSC2-PBS 0.9375<br>WT-LL37 vs. TSC2-LL37 <0.0001<br>TSC2-PBS vs. TSC2-LL37 <0.0001<br>**Day 2 P value**<br>WT-PBS vs. WT-LL37 <0.0001<br>WT-PBS vs. TSC2-PBS 0.9375<br>WT-PBS vs. TSC2-LL37 <0.0001<br>WT-LL37 vs. TSC2-PBS <0.0001<br>WT-LL37 vs. TSC2-LL37 <0.0001<br>TSC2-PBS vs. TSC2-LL37 <0.0001<br>**Day 5 P value**<br>WT-PBS vs. WT-LL37 <0.0001<br>WT-PBS vs. TSC2-PBS 0.9376<br>WT-PBS vs. TSC2-LL37 <0.0001<br>WT-LL37 vs. TSC2-PBS <0.0001<br>WT-LL37 vs. TSC2-LL37 <0.0001<br>TSC2-PBS vs. TSC2-LL37 <0.0001<br>**Day 8 P value**<br>WT-PBS vs. WT-LL37 0.9375<br>WT-PBS vs. TSC2-PBS >0.9999<br>WT-PBS vs. TSC2-LL37 <0.0001<br>WT-LL37 vs. TSC2-PBS 0.9375<br>WT-LL37 vs. TSC2-LL37 <0.0001<br>TSC2-PBS vs. TSC2-LL37 <0.0001<br>**Day 12 P value**<br>WT-PBS vs. WT-LL37 >0.9999<br>WT-PBS vs. TSC2-PBS >0.9999<br>WT-PBS vs. TSC2-LL37 <0.0001<br>WT-LL37 vs. TSC2-PBS >0.9999<br>WT-LL37 vs. TSC2-LL37 <0.0001<br>TSC2-PBS vs. TSC2-LL37 <0.0001

Figure 3E	WT: LL37vsControl P<0.0001; LL37: Raptor cKOvsWT P = 0.01;
Figure 4D	WT: LL37vsControl P<0.0001; LL37: Raptor cKOvsWT P<0.0001;
Figure 6D	P<0.0001 (LL37 vs Control); P<0.0001 (LL37 vs LL37+RAPA)
Figure 6F	WT: LL37vsControl P<0.0001; LL37: Raptor cKOvsWT P<0.0001;
Figure 6G (Nfkb1)	WT: LL37vsControl P<0.0001; LL37: Raptor cKOvsWT P<0.0001;
Figure 6G (Nfkb2)	WT: LL37vsControl P<0.0001; LL37: Raptor cKOvsWT P<0.0001;
Figure 6G (Rela)	WT: LL37vsControl P<0.0001; LL37: Raptor cKOvsWT P<0.0001;
Figure 6G (Relb)	WT: LL37vsControl P<0.0001; LL37: Raptor cKOvsWT P<0.0001;
Figure 6J	P= 0.0002;
Figure 7A	P=0.0025 (LL37 vs Control); P=0.0024 (LL37 vs LL37+RAPA)
Figure 7B (Cxcl11)	WT: LL37vsControl P=0.001; LL37: Raptor cKOvsWT P=0.0294;
Figure 7B (Cxcl12)	WT: LL37vsControl P<0.0001; LL37: Raptor cKOvsWT P=0.0022;
Figure 7B (Cc12)	WT: LL37vsControl P<0.0001; LL37: Raptor cKOvsWT P=0.0005;
Figure 7B (Cc13)	WT: LL37vsControl P<0.0001; LL37: Raptor cKOvsWT P=0.0014;
Figure 7C (CCL3)	DMSO: LL37vsControl P=0.0001; LL37: RAPAvsDMSO P=0.0005;
Figure 7C (CXCL9)	DMSO: LL37vsControl P<0.0001; LL37: RAPAvsDMSO P<0.0001;
Figure 7C (CXCL10)	DMSO: LL37vsControl P=0.0016; LL37: RAPAvsDMSO P=0.0045;
Figure 7C (CXCL11)	DMSO: LL37vsControl P=0.0021; LL37: RAPAvsDMSO P=0.0012;
Figure 7D	P=0.006;
Appendix Figure S1B	P< 0.0001;
Appendix Figure S2F	Vehicle: LL37vsControl P<0.0001; LL37: RAPAvsVehicle P<0.0001;
Appendix Figure S2G (Tnfa)	Vehicle: LL37vsControl P<0.0001; LL37: RAPAvsVehicle P<0.0001;
Appendix Figure S2G (Il6)	Vehicle: LL37vsControl P<0.0001; LL37:

	RAPAvsVehicle P=0.0004;
Appendix Figure S2G (Mmp9)	Vehicle: LL37vsControl P=0.0003; LL37: RAPAvsVehicle P=0.0035;
Appendix Figure S2G (Vegf)	Vehicle: LL37vsControl P<0.0001; LL37: RAPAvsVehicle P<0.0001;
Appendix Figure S4B	DMSO: LL37vsControl P=0.0008; LL37: RAPAvsDMSO P=0.0027;
Appendix Figure S4D	DMSO: LL37vsControl P=0.0028; LL37: RAPAvsDMSO P=0.0145;
Appendix Figure S6A (Il1a)	WT: LL37vsControl P=0.0028; LL37: Raptor cKOvsVehicle P<0.0001;
Appendix Figure S6A (Il1b)	WT: LL37vsControl P=0.0022; LL37: Raptor cKOvsVehicle P=0.04;
Appendix Figure S6C (Nfkb1)	Vehicle: LL37vsControl P<0.0001; LL37: RAPAvsVehicle P<0.0001;
Appendix Figure S6C (Nfkb2)	Vehicle: LL37vsControl P<0.0001; LL37: RAPAvsVehicle P<0.0001;
Appendix Figure S6C (Rela)	Vehicle: LL37vsControl P<0.0001; LL37: RAPAvsVehicle P<0.0001;
Appendix Figure S6C (Relb)	Vehicle: LL37vsControl P<0.0001; LL37: RAPAvsVehicle P<0.0001;
Appendix Figure S6D (left)	DMSO: LL37vsControl P=0.0017; LL37: RAPAvsDMSO P=0.0022;
Appendix Figure S6D (right)	DMSO: LL37vsControl P<0.0001; LL37: RAPAvsDMSO P<0.0001;
Appendix Figure S7A (Cxcl10)	Vehicle: LL37vsControl P=0.0028; LL37: RAPAvsVehicle P=0.0016;
Appendix Figure S7A (Cxcl11)	Vehicle: LL37vsControl P=0.04; LL37: RAPAvsVehicle P=0.03;
Appendix Figure S7A (Ccl3)	Vehicle: LL37vsControl P=0.0029; LL37: RAPAvsVehicle P=0.0056;
Appendix Figure S7A (Ccl5)	Vehicle: LL37vsControl P=0.019; LL37: RAPAvsVehicle P=0.022;
Appendix Figure S7AB(CCL3)	P=0.0219;
Appendix Figure S7AB(CCL5)	P=0.0017;
Appendix Figure S7AB(CXCL8)	P=0.031;
Appendix Figure S7AB(CXCL9)	P=0.0147;
Appendix Figure S7AB(CXCL10)	P=0.0045;
Appendix Figure S7AB(CXCL11)	P=0.0068;
Appendix Figure S7AB(CXCR3)	P=0.0208;
Appendix Figure S7AB(CXCR6)	P<0.0001;
